# Automatic Change Detection of Emotional and Neutral Body Expressions: Evidence From Visual Mismatch Negativity

**DOI:** 10.3389/fpsyg.2019.01909

**Published:** 2019-08-23

**Authors:** Xiaobin Ding, Jianyi Liu, Tiejun Kang, Rui Wang, Mariska E. Kret

**Affiliations:** ^1^Psychology Department, Northwest Normal University, Lanzhou, China; ^2^Key Laboratory of Behavioral and Mental Health of Gansu Province, Lanzhou, China; ^3^Cognitive Psychology Department, Leiden University, Leiden, Netherlands; ^4^Leiden Institute for Brain and Cognition (LIBC), Leiden, Netherlands

**Keywords:** affect, emotional body language, visual mismatch negativity, visual processing, electroencephalography

## Abstract

Rapidly and effectively detecting emotions in others is an important social skill. Since emotions expressed by the face are relatively easy to fake or hide, we often use body language to gauge the genuine emotional state of others. Recent studies suggest that expression-related visual mismatch negativity (vMMN) reflects the automatic processing of emotional changes in facial expression; however, the automatic processing of changes in body expression has not yet been studied systematically. The current study uses an oddball paradigm where neutral body actions served as standard stimuli, while fearful body expressions and other neutral body actions served as two different deviants to define body-related vMMN, and to compare the mechanisms underlying the processing of emotional changes to neutral postural changes. The results show a more negative vMMN amplitude for fear deviants 210–260 ms after stimulus onset which corresponds with the negativity bias that was obtained on the N190 component. In earlier time windows, the vMMN amplitude following the two types of deviant stimuli are identical. Therefore, we present a two-stage model for processing changes in body posture, where changes in body posture are processed in the first 170–210 ms, but emotional changes in the time window of 210–260 ms.

## Introduction

The environment surrounding humans is constantly changing. In order to survive and adapt to this changing environment, our perceptual system has the important task of probing and collecting information regarding these changes (Czigler and Pató, 2009). The visual system, as the most significant part of the perceptual system for humans, plays a crucial role in detecting changes in environmental information. However, due to the limitations in the capacity of the human brain (Marois and Ivanoff, 2005), the visual system has developed the ability to automatically process the most relevant stimuli first, that is, those which can be either just novel (for instance, the sudden opening of the mouth of an interaction partner) or emotionally relevant (such as the emerging smile on an interaction partner’s face) (Kovarski et al., 2017).

The neurophysiological mechanisms of automatic change detection by the visual system have been widely explored in terms of the visual mismatch negativity (vMMN) component of event-related potentials (ERPs) (Kimura et al., 2011; Czigler, 2014). The vMMN is usually obtained in passive oddball paradigms and is defined as activity resulting from the subtraction of activity following standard stimulation from deviant stimulation. Previous studies have obtained effective vMMN activity not only in the change of low-level stimulus features such as color (Czigler et al., 2004; Muller et al., 2012), shape (Maekawa et al., 2005), and motion direction (Pazoalvarez et al., 2015) but also in changes in observed facial expressions of emotion (Susac et al., 2010; Astikainen et al., 2013; Kovarski et al., 2017). This suggests that the neural mechanism related to the processing of the two types of changes (both neutral and emotional) by the visual system can be reflected in vMMN.

The ability to identify changes in emotional expressions is of primary importance in social life (Fujimura and Okanoya, 2013; Chen et al., 2016). Therefore, an emotional facilitation effect has often been reported (Ikeda et al., 2013; Carretié, 2014; Hinojosa et al., 2015). However, only a few studies have directly compared emotional deviancy to neutral deviancy to distinguish the detection of emotional and neutral change (Gayle et al., 2012; Vogel et al., 2015; Kovarski et al., 2017). The study by Vogel et al. (2015) provides direct evidence that emotional (fear) vMMN has advantages over neutral vMMN in terms of latency and amplitude. Another study found that emotional (anger) deviants had a more sustained effect than neutral deviants, even though both deviants showed overlap at the early stage (Kovarski et al., 2017). Furthermore, this result may be explained by another study, which reported that the early difference waves (around 130 ms) reflect the processing of general rule violation, while the later difference waves (around 170 ms) reflect emotional processing (Astikainen et al., 2013). Together, although the detection of emotional deviancy seems to boost vMMN activity, prior to that, there seems to be a stage that just detects deviancy, independent of emotion. However, more research is needed to verify this presumption. Bodily expressions provide an excellent opportunity to do so, as changes in action or emotion lead to a different configuration of the limbs and thus greater changes than in facial expressions (where the position of the eyes, nose, and mouth do not change too much).

Humans express their emotions through various modalities including the voice, the face, and the whole body. Despite the multimodality of emotions, almost all previous studies involving emotion-related vMMN have used facial expressions of emotion as stimulus materials. However, the other important carrier of visual emotional information, body language, has been neglected to a large extent (de Gelder, 2009; Kret et al., 2013). Although previous studies have found that static bodily expressions can be automatically processed, similar to facial expressions (van Heijnsbergen et al., 2007; Gu et al., 2013), the mechanisms of the detection of changes in body expression have not been explored fully. Questions such as whether changes in emotional information contained in body posture can be processed quickly, whether the processing of body expressions is identical to the processing of facial expressions, and whether postural changes are similarly reflected in vMMN as is the case for facial expressions, remain unanswered. Some body postures (such as fear) are signals of approaching danger; preparing for adaptive behavior by rapid and accurate detection of the sudden postural changes in an observed other, is important for survival (de Gelder et al., 2004; de Gelder, 2006, 2009). Research suggests that the processing of body expressions relies on similar visual processing mechanisms as that of facial expressions, both in regard to their timing, as evidenced from electroencephalography (EEG) studies (Stekelenburg and de Gelder, 2004; Meeren et al., 2005; Thierry et al., 2006; Righart and de Gelder, 2007; van Heijnsbergen et al., 2007; Gu et al., 2013), and their location (neural overlap has been demonstrated with fMRI: Giese and Poggio, 2003; Hadjikhani and de Gelder, 2003; de Gelder et al., 2004; Van de Riet et al., 2009; Kret et al., 2011) or both (Meeren et al., 2013). Therefore, we speculated that a change in body posture can be automatically detected and processed, especially when the change reflects a threat signal, and vMMN [expression-related vMMN (EMMN)] could be evoked by this affective processing, in a similar manner as that observed in facial expression studies. In addition, this vMMN activity might have the same distribution as that of vMMN caused by facial expressions.

The N170 is an ERP component that reflects the fast processing of configural information of both faces and bodies (Gliga and Dehaene-Lambertz, 2005). Although body postures can also induce an N170 response, their emotional content does not modulate its amplitude, suggesting it is mainly involved in the structural coding procedure of body expressions (Stekelenburg and de Gelder, 2004; van Heijnsbergen et al., 2007). Another component, the N190, has been considered to be sensitive to both body movements and emotional information as indicated by some previous studies (Borhani et al., 2015, 2016). The component has been widely tested in several studies (Thierry et al., 2006; Righart and de Gelder, 2007; Borhani et al., 2015), and the latency of N190 is often about 50 ms later than N170. We expected that the differences for the two types of change information expressed by the body would be reflected in the N190 component rather than the P1 component, which is sensitive to low-level spatial and physical features of the stimulus (Hillyard and Anllo-Vento, 1998). Therefore, in this study, we selected a body expression without facial information as the stimulus to evoke the N190 component (Thierry et al., 2006), and hypothesized that the N190 amplitude would be enhanced by fear deviants compared with neutral deviants. The present study also attempted to verify body-related vMMN in the occipito-temporal areas, which are usually used for obtaining vMMN in facial studies. On this basis, vMMN evoked by neutral and emotional deviants were compared to explore the specific processing of emotional change detection. If the exploration of emotional changes originates from the simultaneous activation of a general visual change detection mechanism and emotional processing, the neutral and emotional vMMN should partially overlap. Conversely, there may be a special system for emotional change processing.

## Materials and Methods

### Participants

Twenty-eight healthy students participated in this experiment. The EEG data of seven subjects were excluded from the final analysis because of poor behavioral performance (the accuracy rates of task execution were less than 0.8). The final sample consisted of 21 subjects (mean age = 22.38 years, SD = 2.56 years, 11 females). We followed previous studies to base our sample size on. To verify, we performed a power analysis (*f* = 0.2, *α* = 0.05, power = 0.80) by G*Power 3.1.9.3 (Faul et al., 2007, 2009) and found that at least 12 participants were needed to be able to replicate previous studies. Our sample size clearly met this requirement.

All subjects had normal or corrected-to-normal vision and one of them was left-handed. Written informed consent was obtained from each participant after the nature of the experiment had been fully explained. The study was approved by the ethical committee board of the Northwest Normal University.

The participants in this study were engaged in the experimental task and had normal emotion processing abilities, as demonstrated by their high accuracy rates (mean accuracy = 0.914; *SD* = 0.047, range 0.80–0.98).

### Stimuli and Procedure

**Figure 1:**
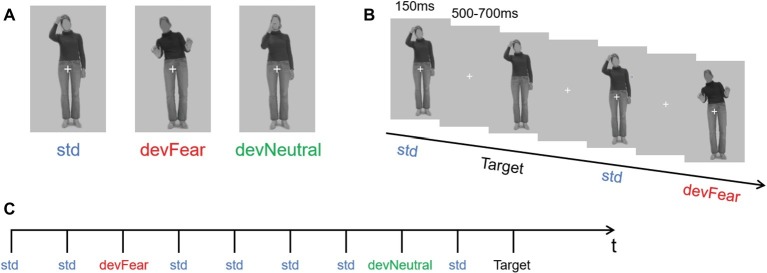
**(A)** shows the three types of stimuli (neutral standard: std; emotional deviant: devFear; and neutral deviant: devNeutral). **(B)** is a task schematic of the oddball sequence. **(C)** shows the time course of the stimulus presented for 150 ms, followed by the cross on a gray screen displayed for 500–700 ms; SOA = 650–850 ms, and a target stimulus (neutral standard without cross, participants need to press the “k” button when it appears). (Statement: the stimuli used in the picture has been approved for publication by the owner).

A different group of 30 participants (mean age = 23.37, age range = 22–27 years old) validated the stimulus materials. They were asked to categorize the stimuli in terms of emotional content (choose from angry, fear, happy, and neutral) and to provide an emotional arousal rating (scale 1–7) for each stimulus. For this validation study, 18 body pictures of six different actresses were selected from the BEAST stimulus set (de Gelder and van den Stock, 2011). Based on this validation study, one identity (female) was selected as the final stimulus (that is, the one with the highest accuracy). The photograph showed an implied motion posture without emotional content (right hand on head) and was presented as the standard stimulus (std); another neutral body posture of the same woman (right hand in front of the mouth), in which motion was also implied, was presented as the neutral deviant stimulus, and an implied motion posture expressing emotion (fear) was presented as the fear deviant stimulus (Figure 1A) (We have obtained the written informed consent of the model). The accuracy of the std. image was 83% and the mean arousal rating was 2.77. Accuracy of the devNeutral and devFear images was 83 and 97%, respectively, and the mean arousal ratings for the two images were 3.60 and 4.20, respectively.

In the formal experiment, the stimuli were presented in an oddball sequence. The probability of occurrence for the three types of stimuli was as follows: standard stimulus (std) *p* = 0.80; neutral deviant stimulus (devNeutral) *p* = 0.10; and fear deviant stimulus (devFear) *p* = 0.10 (Figure 1B).

Participants sat comfortably in an armchair at a distance of 60 cm from a computer monitor. Stimuli were presented and controlled using E-prime software. Each body posture image subtended a visual angle of 1.6° horizontally and 2.6° vertically. Stimulus duration was 150 ms, and the stimulus onset asynchrony (SOA) was randomized between 500 and 700 ms (Figure 1C). The whole sequence comprised 1,280 stimuli; thus, our experiment needed around 15 min to display all the images (for similar procedures, see for instance, Kovarski et al., 2017). The subjects were allowed to have a break after they finished 320 trials. As in previous vMMN studies, subjects were asked to perform a concurrent visual task in order to study automatic change detection. They were required to focus on the white fixation cross in the middle of the picture, and press the “k” button as quickly as possible if the cross disappeared. Thus, the target stimuli were the same as the std. images but did not have a white cross, and the probability of occurrence of the target stimuli was *p* = 0.05. Subjects were allowed to respond both during the stimuli presentation and during the blanks after the stimuli, both instances could generate correct responses.

### Electroencephalography Recording and Processing

EEG signals were recorded using a 64-channel amplifier ANT Neuro EEGO mounted on an electrode cap according to the 10–20 system. Blinks and eye movements were recorded bipolar from the outer canthi of the eyes (horizontal electrooculogram [EOG]) and from above and below the subject’s left eye (vertical EOG). All electrodes were online referenced to the CPz. The impedance of all electrodes was kept below 5 kΩ. The EEG signal was digitized at a sampling rate of 500 Hz.

Subsequent data analyses were carried out off-line using EEGLAB 14.1.2 (Delorme and Makeig, 2004) in the Matlab 9.2.0 development environment (The Mathworks, Natic, MA, USA). The original EEG signals were re-referenced to the common average potential, following which a high pass filter of 0.10 Hz and a low pass filter of 30 Hz were applied to the continuous data. For all three types of stimuli (the target trials were excluded from the analysis), epochs of 600 ms (−100 ms pre-stimulus and 500 ms post-stimulus) were extracted from the continuous EEG signals and were baseline corrected (pre-stimulus activity, from −100 to 0 ms) (van Heijnsbergen et al., 2007; Stefanics et al., 2012). Individual epochs with voltage values exceeding ±50 μV on any channel were rejected from the analysis and ocular artifacts were removed by applying Independent Component Analysis (Delorme and Makeig, 2004) as implemented in EEGLAB. The remaining epochs were averaged separately for each participant and each stimulus type. For each stimulus of interest the average number of artifact-free trials was: 675 ± 127 (std), 89 ± 17 (devFear), and 88 ± 17 (devNeutral).

### Behavioral Data Analysis

Since the task during the experiment was unrelated to the purpose of our study, only the accuracy rate of the target stimulus was calculated, which was used as an indicator of subject’s level of engagement in the experiment.

### Statistical Analyses of Event-Related Potentials

P1 and N190 components were measured on the ERPs evoked by each stimulus type (std, devNeutral, and devFear). Based on previous findings with regard to the P1 and N190, and combined visual inspection of the grand-average waveforms, we chose a time window of 90–150 ms post stimulus presentation to quantify the mean amplitude of the P1 potential, and a time window of 160–220 ms post stimulus presentation to quantify the mean amplitude of N190 (Thierry et al., 2006; Borhani et al., 2015). In addition, the peak amplitude of P1 reached a maximum positive deflection on electrodes O1 and O2. Therefore, electrodes O1 and O2 were chosen as the regions of interest (ROIs) in the P1 analyses, in accordance with previous studies (Righart and de Gelder, 2007; Kovarski et al., 2017). Furthermore, electrodes P7 and P8 were chosen as the ROIs in the N190 analyses on account of the fact that the peak amplitude of this component reached a maximum negative deflection on these two electrodes and also because it was in line with previous studies (Stekelenburg and de Gelder, 2004; Thierry et al., 2006; Borhani et al., 2016).

Both the P1 and the N190 mean amplitudes were analyzed using a repeated-measures analysis of variance (ANOVA) with deviant-type (devNeutral vs. devFear) × hemisphere (left vs. right) as within-subject factors. The effect sizes were calculated in terms of $\eta_{p}^{2}$.

### Statistical Analyses of MMNs

**Figure 2:**
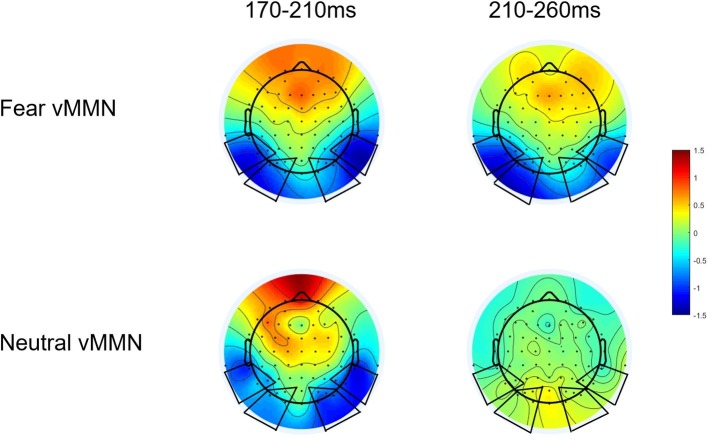
Scalp topographic maps of vMMN (EMMN) components evoked by devFear and devNeutral.

By subtracting the ERPs of the std. stimuli from ERPs of devFear or devNeutral stimuli, an emotion vMMN (fear vMMN) and a neutral vMMN were respectively created to index brain activity specifically elicited by emotional or neutral automatic change detection. According to the topographical maps of these difference waveforms (Figure 2), we selected four ROIs for vMMN amplitude measurements, at left temporal (P7, P5, and TP7), right temporal (P8, P6, and TP8), left occipital (O1, PO3, PO5, and PO7) and right occipital (O2, PO4, PO6, and PO8) electrode clusters. Furthermore, the electrode clusters selected for analyses are marked with black circles in black frames in Figure 2. These ROIs are also consistent with previous studies regarding emotion-related vMMN (Stefanics et al., 2012; Kovarski et al., 2017; Yin et al., 2018). Mean vMMN responses were calculated by averaging across electrodes within the ROIs.

Based on a recent study (Kovarski et al., 2017), we tested vMMN responses for each condition (i.e., fear and neutral deviants) by comparing ERP amplitudes to 0, using Student’s *t*-test corrected for multiple comparisons (Guthrie and Buchwald, 1991) at each ROIs and each time point (Analysis results are presented in Supplementary Materials). This analysis provides preliminary information about the presence of meaningful deflections. And then, visual inspection of the topographical maps (we examined the vMMN topography for every 10 ms intervals between 0 and 500 ms) of these difference waveforms showed larger negativities for deviants relative to standards at occipito-temporal sites in the 170–210 ms intervals for both deviant types. But the fear vMMN is more sustained than the neutral vMMN; it remains activity in the 210–260 ms time window (Figure 2). Combine these reasons, two consecutive time windows were selected for the analysis of vMMN: 170–210 ms and 210–260 ms. This time frames setting is also consistent with the recent study, the early vMMNs latency range corresponding to the common neutral and emotional response, and the following latency range was selected to measure differential activity and investigate specific response to emotional deviants (Kovarski et al., 2017). Within these time-windows ANOVAs were performed including deviant-type (devNeutral vs. devFear) × ROI (temporal vs. occipital) × Hemisphere (left vs. right). When necessary, ANOVA results were corrected following the Greenhouse-Geisser procedure and *post hoc* analyses were corrected with a Bonferroni correction.

## Results

### Results of Event-Related Potentials

**Figure 3:**
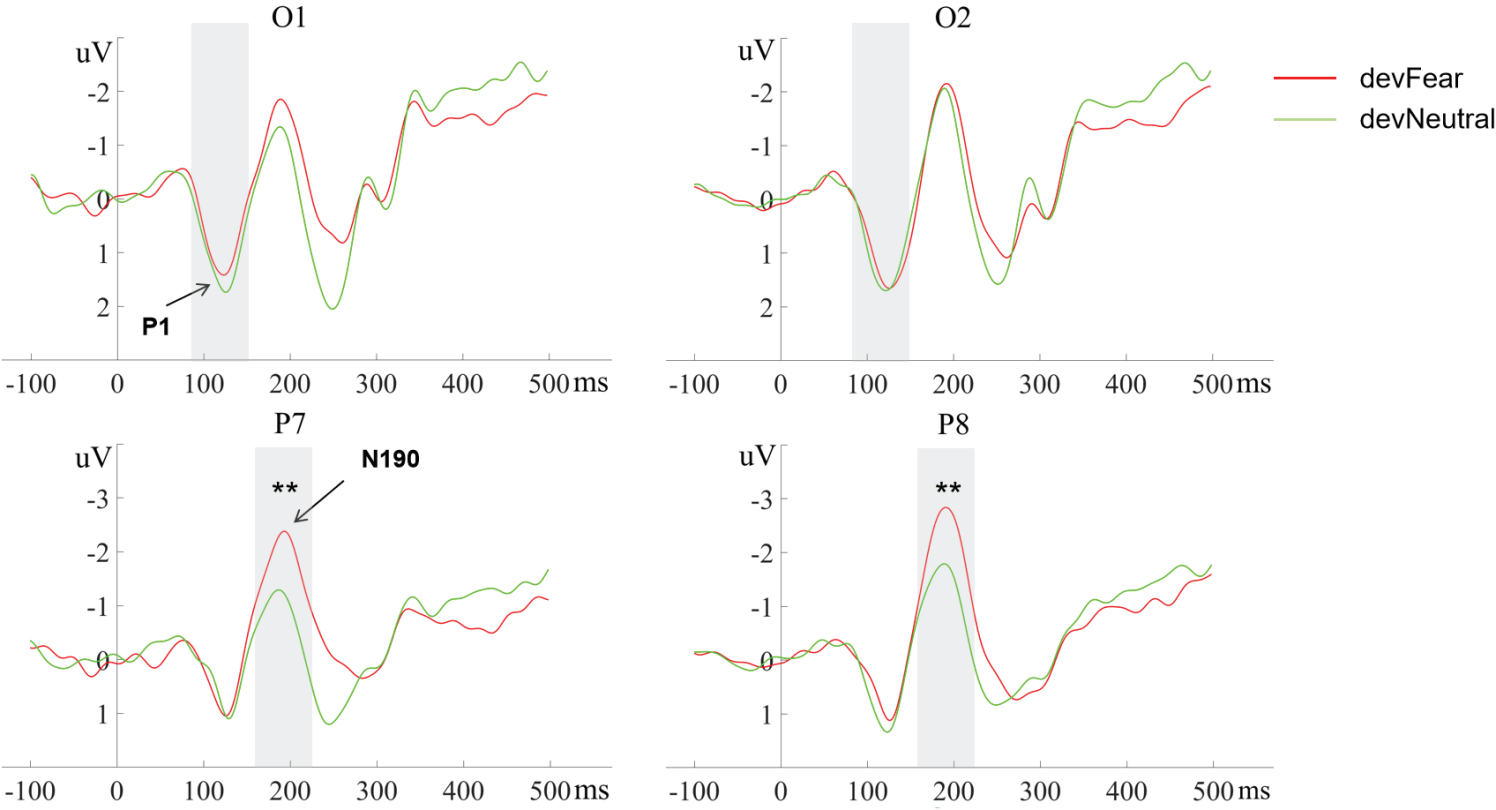
The grand-average ERPs at O1, O2, P7, and P8 evoked by devFear (red line) and devNeutral(green line). ^**^p < 0.01. The P1 was equal following fearful as compared to neutral deviants. The shaded area indicates the statistical analysis time window of the P1 and the N170.

No main effects were observed for deviant-type (*p* = 0.51) and hemisphere (*p* = 0.49) on the P1 amplitude; the deviant-type × hemisphere interaction did not reach significance on this component either (*p* = 0.13), see Figure 3.

The analyses for the N190 amplitude showed a main effect for deviant-type, *F* (1, 20) = 15.93, *p* = 0.001, $\eta_{p}^{2}$ = 0.44. As expected, the N190 amplitudes were more negative in devFear than in devNeutral. Effects of hemisphere (*p* = 0.22) or interaction effects (*p* = 0.65) were not observed, see Figure 3.

### Results of MMNs

**Figure 4:**
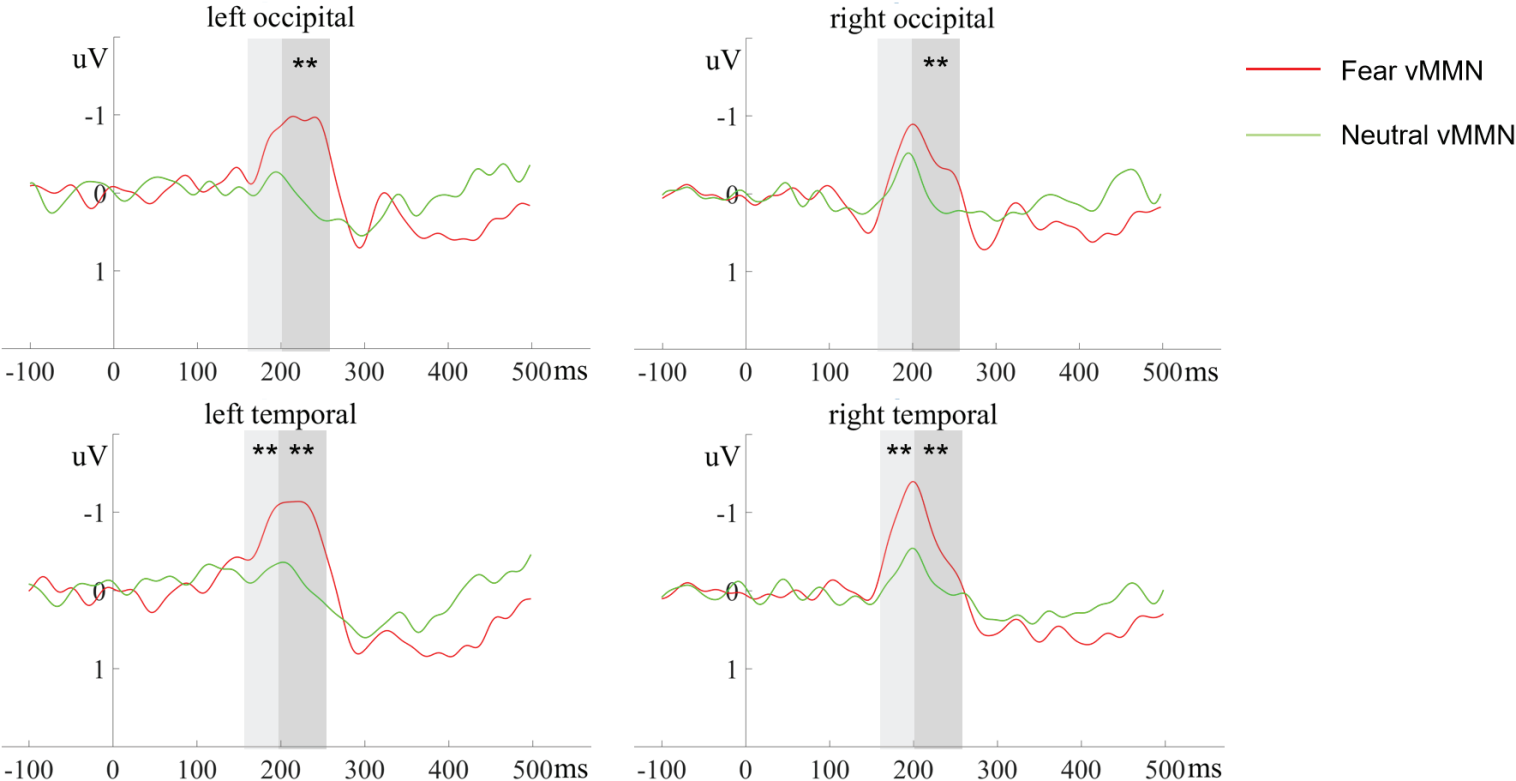
The ERP responses to deviant-minus-standard differential (vMMN) waveforms at different electrode clusters evoked by devFear (red line) and devNeutral (green line). ^**^p < 0.01. The shaded area indicates the statistical analysis time windows of early and late vMMN, respectively.

In the 170–210 ms latency range, a main effect of deviant-type was observed [*F* (1, 20) = 6.72, *p* = 0.017, $\eta_{p}^{2}$ = 0.25], with more negative responses following devFear compared to devNeutral, see Figures 2, 4. A main effect of ROI was also observed [*F* (1, 20) = 7.09, *p* = 0.015, $\eta_{p}^{2}$ = 0.26], with larger amplitudes over the temporal than the occipital sites. Importantly, a significant interaction between deviant-type and ROI was found [*F* (1, 20) = 5.17, *p* = 0.034, $\eta_{p}^{2}$ = 0.21]. Pairwise comparisons indicated that devFear elicited enhanced vMMN amplitudes compared to devNeutral on the temporal sites (*p* = 0.004). By contrast, the type of deviant stimulus did not have a significant effect on the occipital sites (*p* = 0.083), suggesting similar processing of both deviants in this brain region. There was also a significant three-way interaction of deviant-type × ROI × Hemisphere, *F* (1, 20) = 5.04, *p* = 0.036, $\eta_{p}^{2}$ = 0.20. *Post hoc* comparisons revealed that fear vMMNs were more negative on temporal sites than occipital sites (*p* = 0.003). These results were significant over right hemisphere electrodes only. No other main effect (hemisphere) or interactions were significant (*p*s ≥ 0.05).

In the 210–260 ms latency range, the analysis of the vMMN amplitude showed a main effect of deviant-type [*F* (1, 20) = 11.23, *p* = 0.003, $\eta_{p}^{2}$ = 0.36], which was caused by more negative responses to devFear compared to devNeutral, see Figures 2, 4. Moreover, the interaction between deviant-type and Hemisphere was significant [*F* (1, 20) = 6.07, *p* = 0.023, $\eta_{p}^{2}$ = 0.23]. *Post hoc* analysis revealed a larger amplitude over left hemisphere electrodes compared to right hemisphere electrodes for fear vMMN only (*p* = 0.021). The main effect of the ROI and hemisphere was not significant (*p* = 0.08; *p* = 0.11, respectively), and there were no other interactions that were statistically significant (*p*s ≥ 0.05).

## Discussion

The ability to identify changes in emotional expressions is not just important for understanding others’ mental states, but also for avoiding interpersonal conflicts and constructing interpersonal harmony. Although changes in body posture can reveal critical information about emotions, the mechanism behind the processing of these changes has not yet been well explored (Aviezer et al., 2012; for a review, see de Gelder et al., 2010). This is the first study to investigate the automatic processing of changes in observed emotional body language and changes in neutral bodily actions and to test the specificity of the neural mechanism that underlies the processing of emotional deviants during an oddball paradigm. The results first demonstrate a difference between the N190 amplitude in response to emotional compared to neutral deviants. No such difference was observed on the P1. Second, we demonstrate vMMN activity when participants observed changes in both emotional and neutral postures. Third, on several locations and specifically during the later time windows, the emotion-induced vMMN was more sustained and had a larger amplitude than the vMMN that followed neutral images. These findings are consistent with our hypotheses. In the sections below, we discuss these three core findings in the context of the existing literature and present a novel two stage model of processing changes in body posture.

In the current study, the P1 amplitude was not modulated by emotional information. Consistent with our results, a previous study that used face-body compounds as well as isolated faces and bodies, found that although the P1 did not discriminate between anger and fear expressed in isolated faces and bodies, the amplitude of this component was enhanced for incongruent face-body compounds (Meeren et al., 2005). Another study, which used isolated bodies only, presented somewhat different results; the fearful body expression evoked a larger P1 amplitude than the neutral body expression (van Heijnsbergen et al., 2007). Importantly, a recent vMMN study found more negative P1 responses to deviant fearful compared to standard fearful faces (Stefanics et al., 2012). However, in this latter study, it is important to note that no neutral images were used as was the case in the current study. In the previous study, in one block, fearful facial expressions were presented as frequent standards and happy facial expressions as rare deviants. The standard and deviant emotions were swapped during the second block. Thus, the authors used the exact same emotional faces twice, once as standard and once as deviant stimuli, and hence, the difference in P1 amplitude is not caused by facial emotion information (Stefanics et al., 2012). In fact, we can see that the relative novel (unexpected) stimuli in the above studies caused a larger P1 amplitude (Meeren et al., 2005; van Heijnsbergen et al., 2007; Stefanics et al., 2012). Therefore, combining our results and the existing literature, it can be concluded that the P1 reflects the notification of novel or unexpected stimuli rather than the regulation of emotional information. Thus, the more unique a stimulus is, the more likely it is to boost processing at this stage. To conclude, this component represents an initial filtering function before the automatic processing of body expression information.

Another key finding of our study is the main effect of deviant-type on the N190 amplitude, showing that fear deviants induced a larger N190 amplitude than neutral deviants. This result confirms earlier research and shows that emotional stimuli are prioritized at this stage (Borhani et al., 2015). Importantly, using a different paradigm, that study also found that the N190 was sensitive to both neutral (instrumental actions) and emotion information conveyed by body posture. This is in line with the present study, and indicates that this component is sensitive not only to static neutral and emotional body postures, but also to the changes of non-emotional movement information and emotional information reflected in body posture.

The robust vMMN in the time window of 170–260 ms caused by emotional deviants at the occipital and temporal electrodes, supports our hypothesis about the existence of body expression vMMN, and supplements the research gap with regard to the automatic processing of changes in body posture. Some previous facial expression related vMMN studies found that the time range of vMMN corresponds well to the N170 (Chang et al., 2010; Stefanics et al., 2012), and according to the source analyses of the N170, Stefanics et al. (2012) propose the superior temporal gyrus and sulcus as a potential source of the emotional vMMN response. In the present study, the time window of the vMMN we obtained is consistent with the time window of the N190, so we speculate that body expression related vMMN reflects N190 activity. Then, there is an interesting but unexpected finding that the vMMN obtained in the present study has a shorter duration (90 ms) compared to the facial vMMN obtained in past studies (245 ms, Zhao and Li, 2006; 275 ms, Gayle et al., 2012; 105 ms, Kimura et al., 2012; 320 ms, Stefanics et al., 2012). Although there are no previous studies we can refer to compare the duration of body vMMN, we considered two potential explanations for the shorter duration of the vMMN in our study, compared to the studies using facial expressions. First, the most straightforward explanation is that the duration of body vMMN activity is shorter than the duration of facial vMMN activity. Further research is needed to verify whether the duration we observed is indeed specific for bodily expressions or whether for some other reason the duration in the current study was shorter compared to previous studies. Another point of consideration is the fact that in contrast to the previous facial expression studies where a neutral, inactive face served as the standard stimulus, we did not use a completely static neutral body posture as the standard stimulus, but instead used another neutral bodily action. Previous research has shown that the brain is in a higher state of activation when observing a body with implied action than when just observing a static body posture (Grèzes et al., 2007; Kret et al., 2011; Borgomaneri et al., 2015). For this reason, if we had used a calm body posture as standard stimulus, we might have gotten a duration of vMMN similar to previous facial expression studies. That is, going from a calm state to an active one might take longer than going from action into another type of action. In real life, emotional expressions almost never emerge out of a completely neutral inactive state, but rather emerge when we are involved in some kind of action. For that reason, we believe that our study has higher ecological validity and this issue is not only relevant for processing bodily expressions but also for expressions from other modalities including the face or even voice.

In the first time window (170–210 ms), both deviants evoked a significantly negative difference waveform (vMMN). Within this time window, the topographic maps of the vMMN responses for fearful and neutral deviants are remarkably similar (Figure 2), and there was no significant difference between the amplitude of these two vMMN responses at the occipital electrodes; this may have occurred corresponding to the common neutral and emotional change response as suggested in previous studies (Astikainen et al., 2013; Kovarski et al., 2017). Kovarski et al. (2017) demonstrated similar results regarding facial expressions and inferred that the vMMN evoked by either neutral or emotional deviants is based on a general mechanism of visual change processing. Therefore, they suggested that the exploration of emotional changes involves two different pre-attention systems, that is, a mechanism of visual change detection and an additional mechanism of emotion processing. In fact, an earlier study interpreted the early difference wave as a general rule violation detection, while the later difference wave was interpreted as emotional processing (Astikainen et al., 2013). Furthermore, the enhanced vMMN amplitude following fearful body compared to neutral body expressions obtained from this time window (170–210 ms) on the temporal electrodes, suggesting the potential source of the early general mechanism. As mentioned above, the vMMN we obtained reflects the modulating role of the N190 in the automatic processing of changes in body posture, and previous studies have identified the EBA in temporal cortex as the potential neural basis for the N190 (Thierry et al., 2006). While the EBA, which is connected to the amygdala and the parietal cortex, has been considered as a core region of human body perception, it is mainly responsible for visual processing of the body and is sensitive to emotional (Van de Riet et al., 2009; Downing and Peelen, 2011; van den Stock et al., 2012) and motor (Borgomaneri et al., 2015; Downing and Peelen, 2016) information. Thus, the advantage of the vMMN amplitude at temporal electrode clusters may have revealed the active responses of EBA, while similar responses detected at the occipital electrodes may correspond to the general automatic visual processing of body stimulus changes in the Striate Cortex (V1) or Extrastriate Cortex.

More sustained and stronger vMMN responses evoked by fear deviants in 210–260 ms is consistent with the “negativity bias” reported in previous vMMN studies (anger: Kovarski et al., 2017, sadness: Zhao and Li, 2006; Gayle et al., 2012, and fear: Stefanics et al., 2012). Previous studies have shown that negative emotion takes more resources to process compared to positive or neutral emotion, even when attention is limited by high-load tasks (Srinivasan and Gupta, 2010; Gupta and Déak, 2015; Gupta and Srinivasan, 2015; Gupta et al., 2016). Negative emotions were also found to initially capture and hold attention compared to positive and neutral (Ciesielski et al., 2010; Srinivasan and Gupta, 2011). This may partly explain the current findings. However, other studies that compared positive and negative vMMN did not show this effect (Astikainen and Hietanen, 2009; Astikainen et al., 2013), suggesting that differences in protocols (i.e., concurrent task, paradigm, and stimuli) modulate the emotion-related vMMN (Kovarski et al., 2017). In several previous studies, attention is usually directed toward a task presented in a different sensory modality to prevent attentional processes that might overlap with the vMMN component (Zhao and Li, 2006; Astikainen and Hietanen, 2009; Gayle et al., 2012), and this may be a limitation of present study. Without considering the influence of emotional valence, these studies support the specificity of automatic processing of emotional changes, and our results extend the advantageous effect of this processing of emotional changes to the field of body expression.

Fear vMMN showed differential lateralization patterns between the two time windows. In the early period, vMMN responses to fearful deviants were more negative over right temporal sites, but neutral vMMN did not show similar results. This observation is in support of the “right hemisphere hypothesis”, postulating that the right hemisphere is dominant for processing emotions (Borod et al., 1998). Gupta and Raymond (2012) presented a central, irrelevant, expressive (angry, happy, sad, or fearful) or neutral face prior to a letter search task, and found that emotional processing is right hemisphere biased than non-emotional stimuli. Several previous vMMN studies also reported a right-hemisphere lateralization of the emotional vMMN (Gayle et al., 2012; Li et al., 2012; Vogel et al., 2015). Consistent with the above research, the current result confirms that the processing of fearful body change information in this period also has the right hemisphere advantage. By contrast, the hemispheric dominance reversed in the subsequent time window, i.e. increased vMMN amplitudes were observed on the left hemisphere electrode sites for fear deviants within 210–260 ms. This left hemispheric effects may correspond to contextual effects that were observed in an facial related study. This study used face-context compounds stimulus to investigate how the early stages of face processing are affected by emotional scenes, and found that N170 amplitudes were enhanced on the left hemisphere electrode sites when fearful faces were accompanied by fearful scenes rather than happy or neutral scenes (Righart and de Gelder, 2008). The researchers suggested emotions may combine specifically for fear at early stage of encoding. In fact, a large number of vMMN studies have suggested that oddball sequence provides unintentional temporal context (Kimura et al., 2012; Stefanics et al., 2012; Vogel et al., 2015). Therefore, in this processing stage, the fear deviants may be integrated with the background information extracted from the previous stimulus sequence, and the left hemisphere also has the advantage of integrating fear change information. The different activation patterns of hemisphere in the two adjacent vMMN time windows further revealed the different automatic processing stages of body expressions.

**Figure 5:**
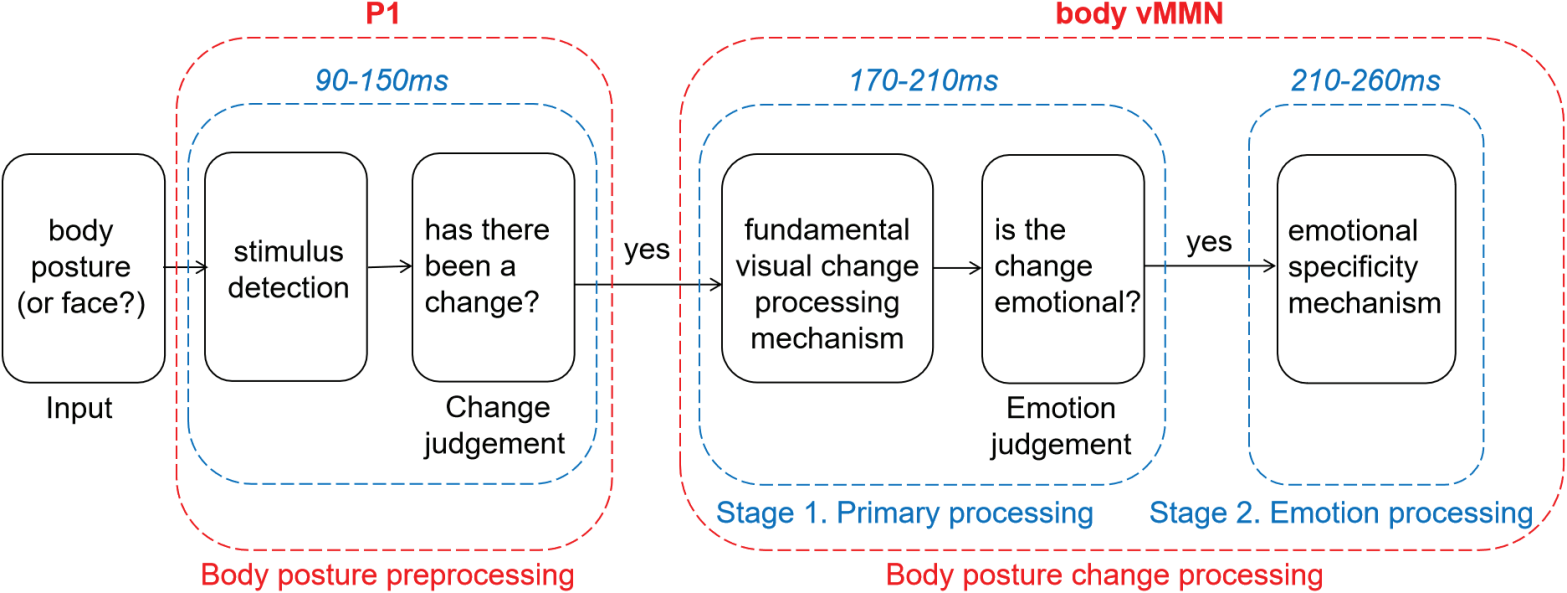
Two-stage model of processing changes in body posture.

In summary, our results about vMMN suggest that emotional and neutral changes in body posture are initially processed by the same automated processing system, and emotional information is then further analyzed. We here propose a two-stage model that is partly based on previous literature (Figure 5). In an earlier study, using emotional expressions, Chang et al. (2010) showed two peaks of vMMN that have been interpreted as two different stages reflecting either modulation of the N170 and P250, and this view was echoed by two other emotional expression vMMN studies (Zhao and Li, 2006; Stefanics et al., 2012). Furthermore, two different stages have been described, reflecting detection and pre-attentional processing in another emotional expression vMMN study (Astikainen and Hietanen, 2009). In line with our results, Kovarski et al. (2017) found similar activity following the neutral and the emotional deviants around the first and second peaks (100–200 ms and 250–350 ms, respectively) and sustained negative activity for angry deviants only in the time windows following the peaks (150–300 ms and 350–480 ms). The authors explain their result as reflecting two distinct preattentional systems: the visual change detection mechanism and an additional emotional processing stage. It provides powerful support for our model and suggests that this model may be applicable to a wider range of emotional carriers. In addition, we have updated this model based on the present study in the following way. First, we added a preprcocessing procedure that always selects novel (unexpected) expressional information into automatic change detection and secondly, we adapted this model to the body N190 activity. Further research is needed to revise and update this model further.

## Conclusions

Before giving the final summary, we want to emphasize the limitation of current statistical method. The statistical data mining in our study is not independent from the main hypothesis well, and should be regarded as an explorative approach. But with limited explanatory power, our study reveals that changes in observed neutral or emotional body postures recruit activity in early automatic processing areas, indexed by vMMN activity, in a slightly different way. It is worth noting that the vMMN evoked by neutral and emotional deviants show two different stages. The first stage is a fundamental visual change processing stage. The second stage is an emotion processing stage (Figure 5). These findings are consistent with previous studies of facial expression. That might imply that the detection of emotional changes expressed through the face or body and possibly through other modalities as well, relies on similar brain mechanisms. Further studies need to verify whether this is indeed the case, in the case of a greater methodological rigor.

## Data Availability

The datasets generated for this study are available on request to the corresponding author.

## Ethics Statement

This study was carried out in accordance with the recommendations of “Ethics of psychological research, The scientific and research Ethics Committee of the School of Psychology, NWNU” with written informed consent from all subjects. All subjects gave written informed consent in accordance with the Declaration of Helsinki. The protocol was approved by the “The scientific and research Ethics Committee of the School of Psychology, NWNU.”

## Author Contributions

JL and XD both contributed to conceive and study design. JL and RW performed experiments and collected data. JL and TK analyzed the behavior and ERP data and drafted the manuscript. MK provided critical revisions. MK and JL approved the final version of the manuscript for submission. All authors read and approved the submitted version.

### Conflict of Interest Statement

The authors declare that the research was conducted in the absence of any commercial or financial relationships that could be construed as a potential conflict of interest.

## Supplementary Material

Supplementary Figures 1–4 vMMN amplitude and *p*-value for *t*-test of four ROIs (*p* < 0.05). The black boxes are the time windows of effective negative component in these images.
