# Supplementary figures and images for: Automatic Change Detection of Emotional and Neutral Body Expressions: Evidence From Visual Mismatch Negativity
Source: Front Psychol. 2019 Aug 23;10:1909. doi: 10.3389/fpsyg.2019.01909 (PMC6716465; doi:10.3389/fpsyg.2019.01909)

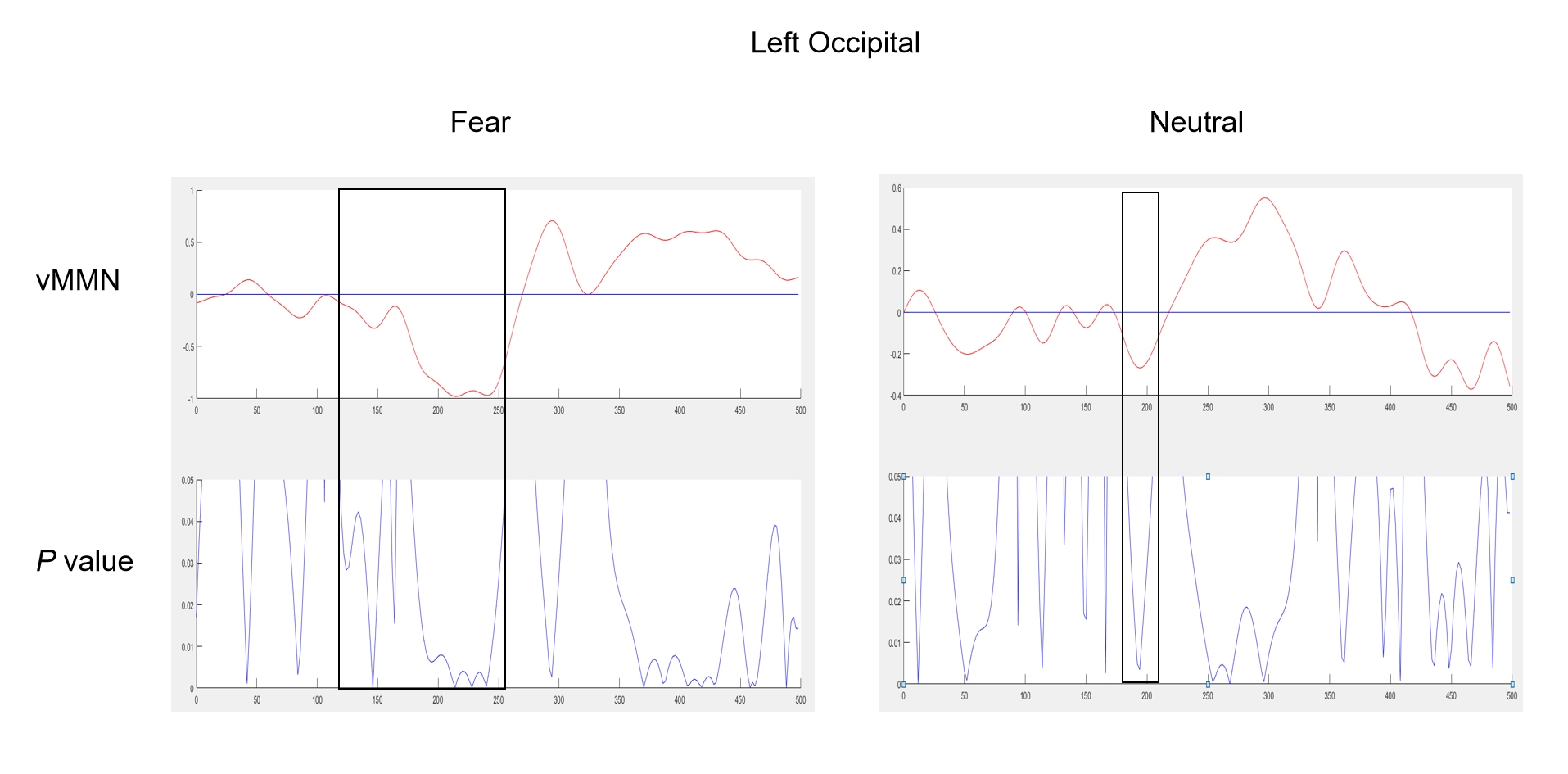

Supplement: Supplementary Figures 1–4 — vMMN amplitude and p-value for t-test of four ROIs (p < 0.05). The black boxes are the time windows of effective negative component in these images. [file Image_1.JPEG]

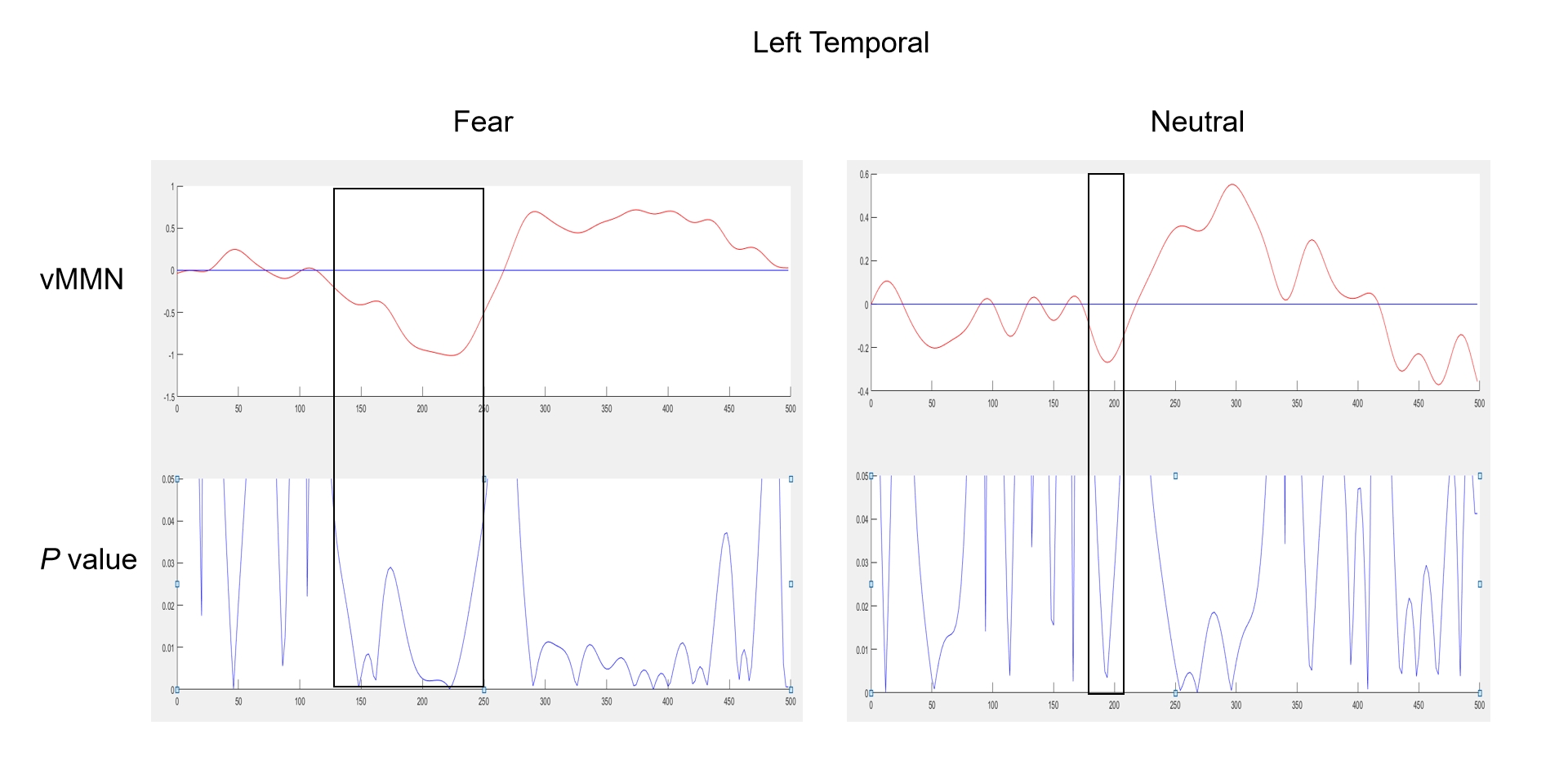

Supplement: Supplementary file 2 [file Image_2.JPEG]

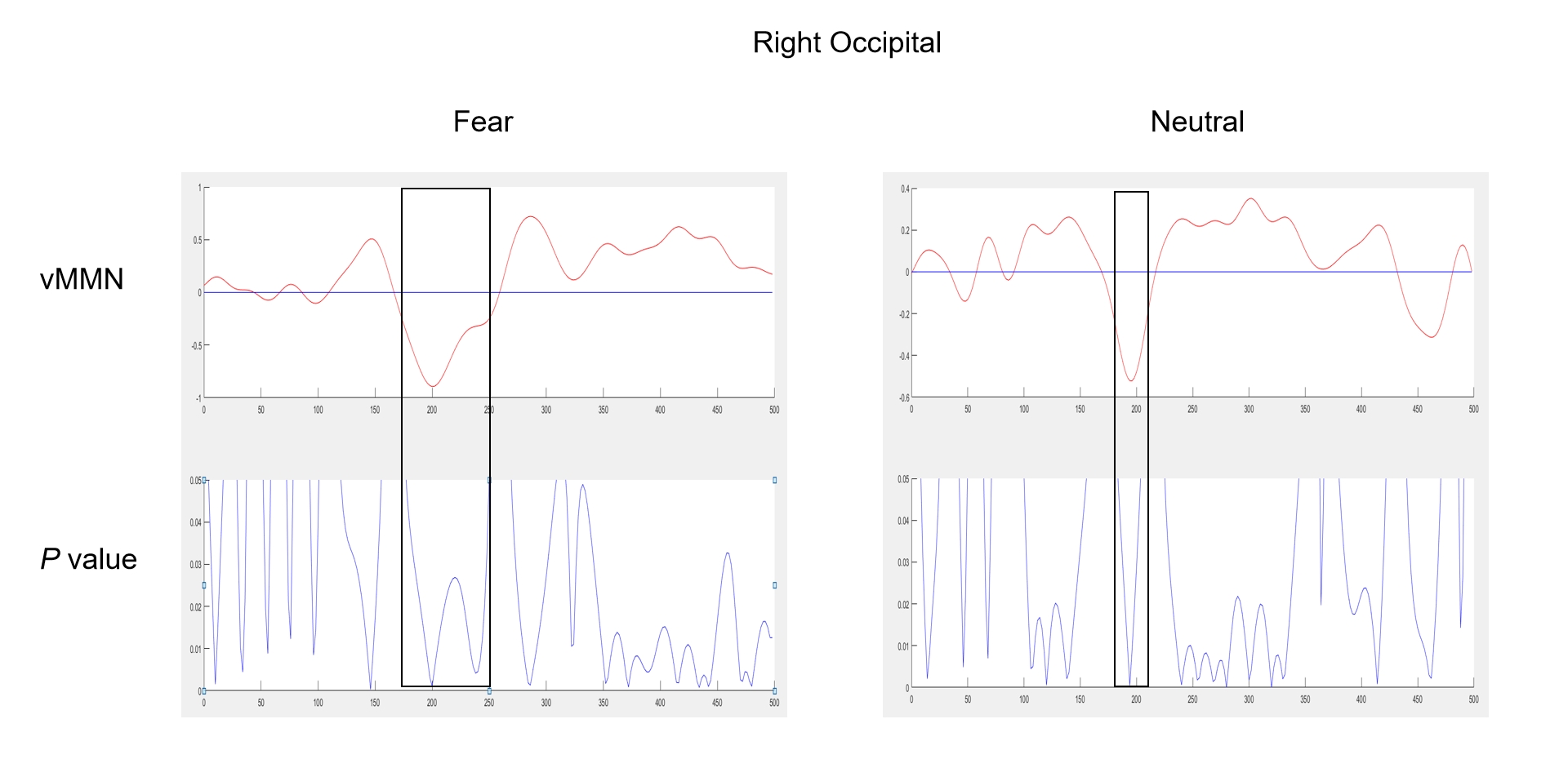

Supplement: Supplementary file 3 [file Image_3.JPEG]

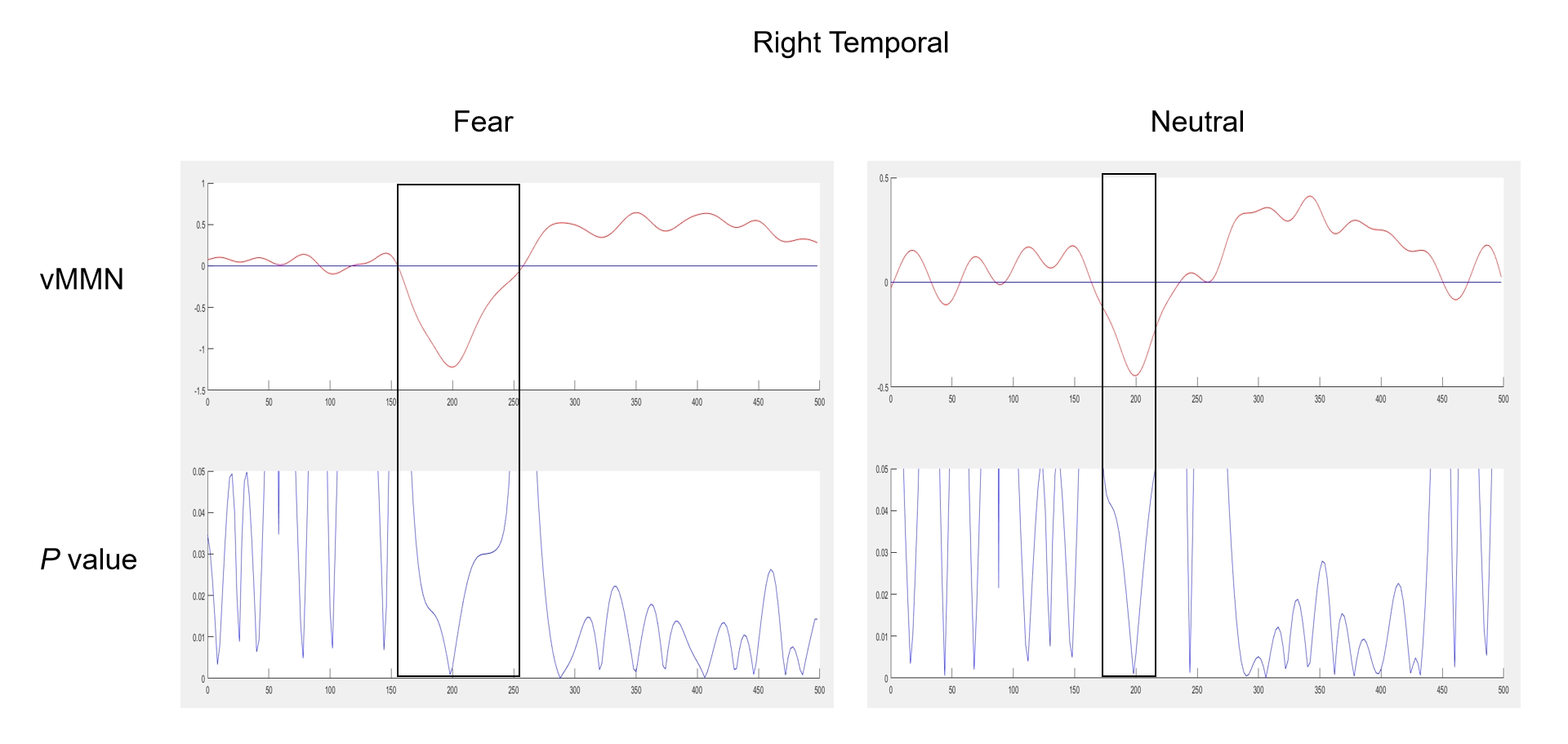

Supplement: Supplementary file 4 [file Image_4.JPEG]
